# Thioflavin T in-gel staining for *ex vivo* analysis of cardiac amyloid 

**DOI:** 10.3389/fmolb.2025.1505250

**Published:** 2025-05-13

**Authors:** Joseph Oldam, Irina Tchernyshyov, Jennifer Van Eyk, Juan Troncoso, Charles G. Glabe, Giulio Agnetti

**Affiliations:** ^1^ Johns Hopkins University School of Medicine, Baltimore, MD, United States; ^2^ Advanced Clinical Biosystems Institute, Cedars-Sinai Hospital, Beverly Hills, CA, United States; ^3^ University of California Irvine School of Biological Sciences, Irvine, CA, United States; ^4^ Department of Biomedical and Neuromotor Sciences (DIBINEM), University of Bologna, Bologna, Italy

**Keywords:** amyloid, mass spectrometry, proteinopathies, desmin, heart failure

## Abstract

There are limited options to quantify and characterize amyloid species from biological samples in a simple manner. Thioflavin T (ThT) has been used for decades to stain amyloid fibrils, but to our knowledge, we were the first to use it in-gel. Thioflavin T in-gel staining is convenient as it is fast, inexpensive, accessible to most laboratories, and compatible with other fluorescent stains and downstream analyses such as mass spectrometry (MS).

## Introduction

Organ and systemic proteinopathies represent one of the main areas of public health concern in Westernized society in this century (Chiti and Dobson, 2009). The toxic nature of pre-fibrillar and fibrillar amyloid is well-established in neurocognitive disease (Mullapudi et al., 2023); however, their role and prevalence are also emerging in cardiovascular diseases (Del Monte and Agnetti, 2014). Most amyloid species are quantified using either fluorescent, radiolabeled, or immunological probes, as well as mass spectrometry (MS) (Seino et al., 2021; Wall et al., 2013; Ruiz-Arias et al., 2022; Iino et al., 2021). From the technological standpoint, MS is arguably one of the techniques with the highest impact on biomedical sciences in this century (Agnetti et al., 2016). Cutting-edge MS-based approaches such as isotope incorporation are currently being used in the study of protein misfolding, but their application is still confined to a few laboratories worldwide (Mackmull et al., 2022). It is our opinion that a simple way to detect and quickly isolate amyloid species *ex vivo*, which is compatible with “classic” MS, would greatly benefit the study of an increasing number of organ-based proteinopathies.

We optimized a straightforward, affordable method to stain amyloid species in-gel and tested it on a well-established model of murine cardiac amyloidosis (Sanbe et al., 2004), a kind gift from Dr. Jeffrey Robbins and others. The idea of an in-gel fluorescent staining for amyloids was inspired by an ingenious study by Hervé et al. (2009) who used thioflavin T (ThT) in combination with a general fluorescent protein stain (SYPRO Ruby) to assess cross-contamination of surgical tools with prions. As SYPRO Ruby is typically used to stain proteins separated by gel electrophoresis, we hypothesized that ThT would also work in-gel. We combined the staining with both a modified version of blue native gels in the presence of SDS—referred to as not-so-native (NSN) gels—and regular SDS-PAGE. These approaches have the advantage of separating oligomers and fibrils while enabling easy quantification of these species in a simple gel format across a wide range of molecular sizes. ThT and other related compounds have an affinity for both fibrils and pre-fibrillar oligomers (Maezawa et al., 2008; Khurana et al., 2005) (named pre-amyloid oligomers, or PAOs henceforth), and its fluorescence increases when ThT is bound to these species (Biancalana and Koide, 2010).

Thioflavin T’s signal can be conveniently acquired using a laser scanner equipped with a Cy2 filter (λ_ex/em_ = 488/520). A Cy5 (λ_ex/em_ = 633/670) filter can be used as a reference filter and for total protein quantification based on Coomassie staining. In short, these aspects, combined with the low cost of the dye, make the described method accessible to a variety of laboratories. The additional optimization of a classical reducing method also allows us to compare the results using ThT with those obtained using more classical approaches [e.g., the A11 antibody (Kayed et al., 2003)]. For these reasons, we believe that this new approach will enable the expeditious quantification, purification, and characterization of amyloid species in different proteinopathies (e.g., cardiac amyloidosis, Alzheimer’s disease (AD), and Parkinson’s disease).

We first used the ThT in-gel stain to validate the amyloid properties of desmin oligomers in a canine model of dyssynchronous heart failure (HF) (Agnetti et al., 2014). In the study, we separated the oligomers under non-reducing conditions and in the presence of sodium dodecyl sulfate (SDS), using a blue native gel format, which we nicknamed NSN.

More recently, we tested the performance of the staining and optimized the protocol for classical (reducing) SDS-PAGE using the most established model of cardiac proteotoxicity (Sanbe et al., 2004; Rainer et al., 2018). The transgenic mice used in this study express a mutated form (R120G) of the most abundant small heat shock protein in the heart: α-B-crystallin (cryAB). The cardiac-specific expression of this mutated chaperone in transgenic mice induces the formation of desmin PAOs and fibrils.

**FIGURE 1:**
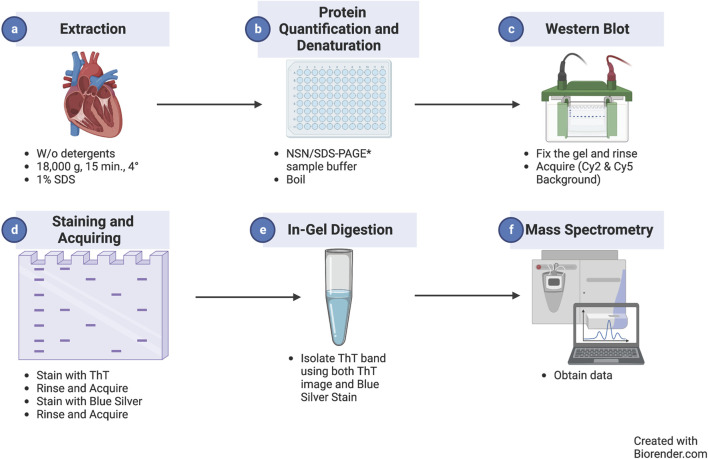
Thioflavin T staining/MS workflow. Diagram representing the workflow of the experimental method introduced in this study starting with protein extraction **(a)** followed by protein quantification/detection **(b)**, western blot to fix the gel **(c)**, staining and acquisition **(d)**, in-gel digestion **(e)**, and mass spectrometry for analysis **(f)**.

In the optimized protocol outlined in Figure 1, cardiac extracts are prepared according to our optimized protocol to separate myofilament and cytosolic-enriched fractions (Rainer et al., 2018). This separation consists of a homogenization step in the absence of detergents (cytosolic-enriched, soluble fraction), followed by centrifugation and re-suspension in the presence of detergents (SDS for the myofilament-enriched, insoluble fraction), similar to the protocols used in neuroscience to enrich for fibrils (Wirths, 2017) (Figure 1A). After protein quantification (Figure 1B), samples are diluted in the proper sample buffer for either NSN-PAGE or classical SDS-PAGE, boiled in the latter case, and separated using a standard 1D-PAGE apparatus (Figure 1C). After separation, the gel, which is already blue in the case of NSN and clear in the case of regular denaturing gels, is fixed, rinsed thoroughly in water, and stained with Coomassie for the reducing gels, and its images are digitized before staining with ThT to ensure that potential protein auto-fluorescence in the ThT (Cy2) channel is accounted for (Figure 1D). The gel is then incubated with 50 µM ThT in acidified water and rinsed several times in acidified water to reduce the background and remove ThT speckles before final image acquisition (Figure 1D, see *Methods* for details). Following image acquisition, the gel can be stored in bi-distilled water or dried. The ThT-positive gel bands can be located using the Coomassie image as a reference and excised for downstream in-gel digestion and MS analysis (Figures 1E, F).

**FIGURE 2:**
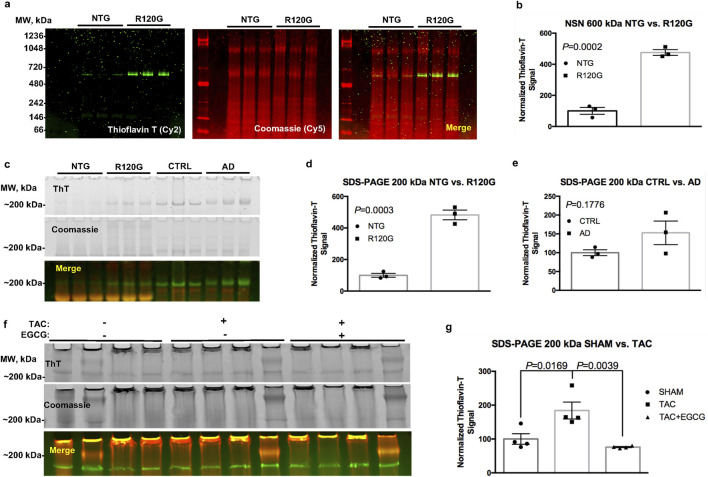
ThT stain in cardiac and brain tissue extracts. Representative image of an NSN gel used to separate fibrillar aggregates from NTG and R120G cryAB mice **(a)**, ThT in green and Coomassie in red; corresponding densitometric analysis of the ThT signal at ∼ 600 kDA is also provided **(b)**. Representative image of a classical SDS-PAGE gel showing a ThT signal at ∼200 kDa in NTG and R120G cryAB mouse cardiac extracts and extracts from healthy human brain (CTRL) and AD human brain tissues from the Baltimore Longitudinal Study on Aging (BLSA) **(c)**. Densitometric analysis of the ThT signal at ∼200 kDA is also provided **(d, e)**, respectively). A representative image of the effects of EGCG on extracts from TAC and sham-operated mouse hearts is also provided **(f)**, along with the respective densitometric analysis **(g)**. NTG, non-transgenic; R120G, R120G cryAB mouse heart extracts; CTRL, healthy human brain tissue; AD, Alzheimer’s disease; EGCG, epigallocatechin gallate; ThT, Thioflavin T. Mean ± SD is plotted in all graphs. *P*-values were obtained using a student’s *t*-test **(b, d–e)** or one-way ANOVA, followed by Sidak’s multiple comparison tests **(g)**. Please refer to the text for the abbreviations.

When this procedure was used to analyze short cardiac amyloid fibrils using NSN-PAGE, we could detect a ∼5-fold increase in a sharp ThT-positive band in R120G cryAB (R120G) mice vs. non-transgenic (NTG) controls (*P* = 0.0002). Signals for both ThT and Coomassie were digitized and contrast-enhanced (Figure 2A) to enable improved visualization and quantification (Figure 2B). Interestingly, the ThT-positive band was detected at an apparent molecular weight of ∼600 kDa. Although NSN separation does not allow for proper molecular weight calibration, we reported a similar electrophoretic mobility of ThT-positive bands in canine failing hearts (Agnetti et al., 2014), suggesting a mechanism of fibrillization that is conserved across species and independent of genetic mutations. This is relevant as many organ proteinopathies (e.g., Parkinson) are sporadic in nature (Del Monte and Agnetti, 2014).

To broaden the applicability of the protocol, we adapted it for use with reducing (classical) SDS-PAGE. We tested the method using both R120G cryAB samples vs. NTG controls and clinical brain specimens from AD patients vs. healthy individuals (CTRL) (Figure 2C). In agreement with our previously published results and the NSN protocol, we detected a ∼5-fold significant increase in a ThT-positive band at ∼200 kDa under reducing conditions in cryAB R120G cardiac extracts vs. NTG controls (*P* = 0.0003; Figures 2D, E). We also detected a ∼1.5-fold increase in a ∼200-kDa ThT-positive band compatible with fibrils in brain extracts from Alzheimer’s patients compared to healthy controls (*P* = 0.176; Figure 2E). We attribute the lack of significance with the latter comparison to the limited number of samples used in this study and the larger variability in patients’ cohorts compared to mice with homogeneous genetic backgrounds. As mentioned, Coomassie post-stain allowed us to cross-reference, excise the ThT-positive bands with the naked eye (Figure 2C, mid panel), and confirm equal loading. Under these settings, we were able to confirm the specificity of the stain using a standardized and more robust separation method (classical SDS-PAGE).

Finally, we used the ThT in-gel protocol to measure the accumulation of desmin-positive amyloid fibrils in an established murine model of HF based on transverse aortic constriction (TAC) vs. sham-operated controls (Rainer et al., 2018). We detected a ∼2-fold increase in a ∼200-kDa ThT-positive band in extracts from TAC mouse samples vs. controls (*P* = 0.0169; Figure 2G).

To further validate the specificity of ThT staining for amyloid aggregates, we exploited the ability of the small-molecule epigallocatechin gallate (EGCG) to reduce or reverse fibrillization in prion strains (Roberts et al., 2009). Notably, treating cardiac extracts with 100 µM EGCG for 30 min at room temperature (RT) reversed the increase in ThT observed in TAC samples to control levels (*P* = 0.0039) (Figure 2G). This observation supports the specificity of ThT staining for amyloid species. These combined data suggest that ThT in-gel staining could be applied to a wide number of proteinopathies, including those afflicting organs other than the heart (e.g., the brain).

## Methods

### Tissue procurement

Eight- to twelve-week-old C57BL/6 mice were subjected to TAC or sham surgery through the Cardiac Physiology Core at Johns Hopkins, as previously described (Rainer et al., 2018; Oeing et al., 2021). Four weeks after surgery, TAC mice developed overt HF, as indicated by a decrease in fractional shortening (≤40%) measured by echocardiography. At that time point, mice were anesthetized, and the heart was harvested and snap-frozen according to the protocol approved by the local IACUC. In brief, mice were anesthetized using isoflurane. Upon reaching deep anesthesia with isoflurane (absence of the toe pinch reflex), cervical vertebrae were dislocated, and the heart was quickly extracted through thoracotomy. Freshly explanted hearts were quickly rinsed in ice-cold PBS containing protease (cOmplete Mini, Roche) and phosphatase inhibitors (PhosSTOP, Sigma) to remove excess blood, gently blotted on the clean filter paper to remove excess liquid, snap-frozen in liquid nitrogen, and stored at −80C until further processing. Frozen ventricles from R120G cryAB mice were kindly provided by Dr. Jeff Robbins at the Cincinnati Children’s Hospital. Frozen brain tissue from Alzheimer’s patients and healthy controls was kindly provided by DR. Juan Troncoso as part of the Baltimore Longitudinal Study on Aging at Johns Hopkins University School of Medicine. All frozen tissue samples were stored at −80 °C until ready for protein extraction.

### Protein extraction

Thirty to fifty milligrams of frozen tissue were homogenized in cold PBS (Gibco), supplemented with protease (cOmplete Mini, Roche) and phosphatase (PhosSTOP, Sigma) inhibitors and 25 mM HEPES (AMRESCO). Pre-weighed 2-mL vials were used to obtain the tissue weight. A dry ice-cooled metal bead (Retsch) was placed in each vial, along with five volumes (V/W) of homogenization buffer. Samples were then homogenized for 2 min at 28 Hz using a bead mill (MM400, Retsch) and cooled racks. After milling, samples were pulse-centrifuged and placed in a magnetic rack (Invitrogen) to ensure that the metal bead remained on the side of the vial, and the homogenate was transferred to a new clean vial on ice. The beads were rinsed with three volumes of ice-cold homogenization buffer (V/W) under vortexing, and the resulting solution was combined with the respective homogenate. The resulting tissue homogenates were centrifuged for 15 min (18,000 rcf, 4°C) to obtain soluble and insoluble fractions, which were separated, snap-frozen, and stored at −80°C until further use.

Soluble and insoluble fractions were denatured in LDS buffer (Thermo) within the homogenization buffer, supplemented with 1% DTT (V/V), followed by heat denaturation at 95°C for 10 min. Protein concentration in the denatured samples was measured using the EZQ Protein Quantification Kit (Thermo).

### Electrophoresis

For NSN gels, protein extracts from the insoluble fraction were diluted in blue native sample buffer (25 mM Bis-Tris, Bio-Rad; 0.015 ml of 1 N hydrochloric acid, Fisher Scientific; 10% glycerol, Sigma Aldrich; 25 mM NaCl, Sigma Aldrich; 0.001% Ponceau S, Fisher Scientific; 2% SDS, Fisher Scientific). After incubating samples at RT for 30 min, they were clarified by centrifugation (18,000 rcf, 30 min, 4°C) to remove the insoluble material, and the resulting supernatant was supplemented with 0.25% Coomassie Blue G-250 (Fisher Scientific). A measure of 40 μg of protein per sample was then separated using NuPAGE pre-cast native gels (Thermo) for NSN, while 20 µg of protein were used for classic SDS-PAGE using 3%–12% NuPAGE gels (Thermo). Both types of electrophoreses were performed at 150 V for approximately 1 h. After separation, gels were fixed in 10% acetic acid (Fisher Chemical), 40% methanol (Fisher Chemical), and bi-distilled water overnight to remove excess SDS for downstream excess analysis.

### Gel staining and acquisition

The following day, gels were rinsed several times in bi-distilled water to remove excess methanol, and the background fluorescent signal for ThT and Coomassie (Cy2 and Cy5 filters, respectively) was acquired using a Typhoon laser scanner (GE). Gels were subsequently stained with 0.1% (W/V) ThT in acidified water [13.8 µL of 1 N hydrochloric acid (Fisher Scientific) per 50 mL of bi-distilled water] for 1 h in the dark. The ThT working solution was prepared at least 1 hour in advance to maximize solubilization and was kept in the dark. After ThT staining, gels were rinsed extensively in acidified water to remove speckles, followed by a new acquisition of the Cy2 and Cy5 signals using the Typhoon. Following ThT signal acquisition, gels were stained with blue silver (Candiano et al., 2004) for ≥20 min at RT, rinsed extensively in acidified water, and imaged again to acquire the fluorescent signal for Coomassie (Cy5 filter). The resulting merged images of Coomassie and ThT signals (Figure 2) were used for quantitative analysis and to locate the ThT-positive bands with the naked eye using Coomassie as a reference.

### Mass spectrometry

Please refer to the online Methods and Results for LC-MS analysis (Supplementary Figures 1, 2).

## Data Availability

The original contributions presented in the study are included in the article/Supplementary Material; further inquiries can be directed to the corresponding author.
