# Supplementary figures and images for: Thioflavin T in-gel staining for ex vivo analysis of cardiac amyloid
Source: Front Mol Biosci. 2025 May 13;12:1505250. doi: 10.3389/fmolb.2025.1505250 (PMC12106040; doi:10.3389/fmolb.2025.1505250)

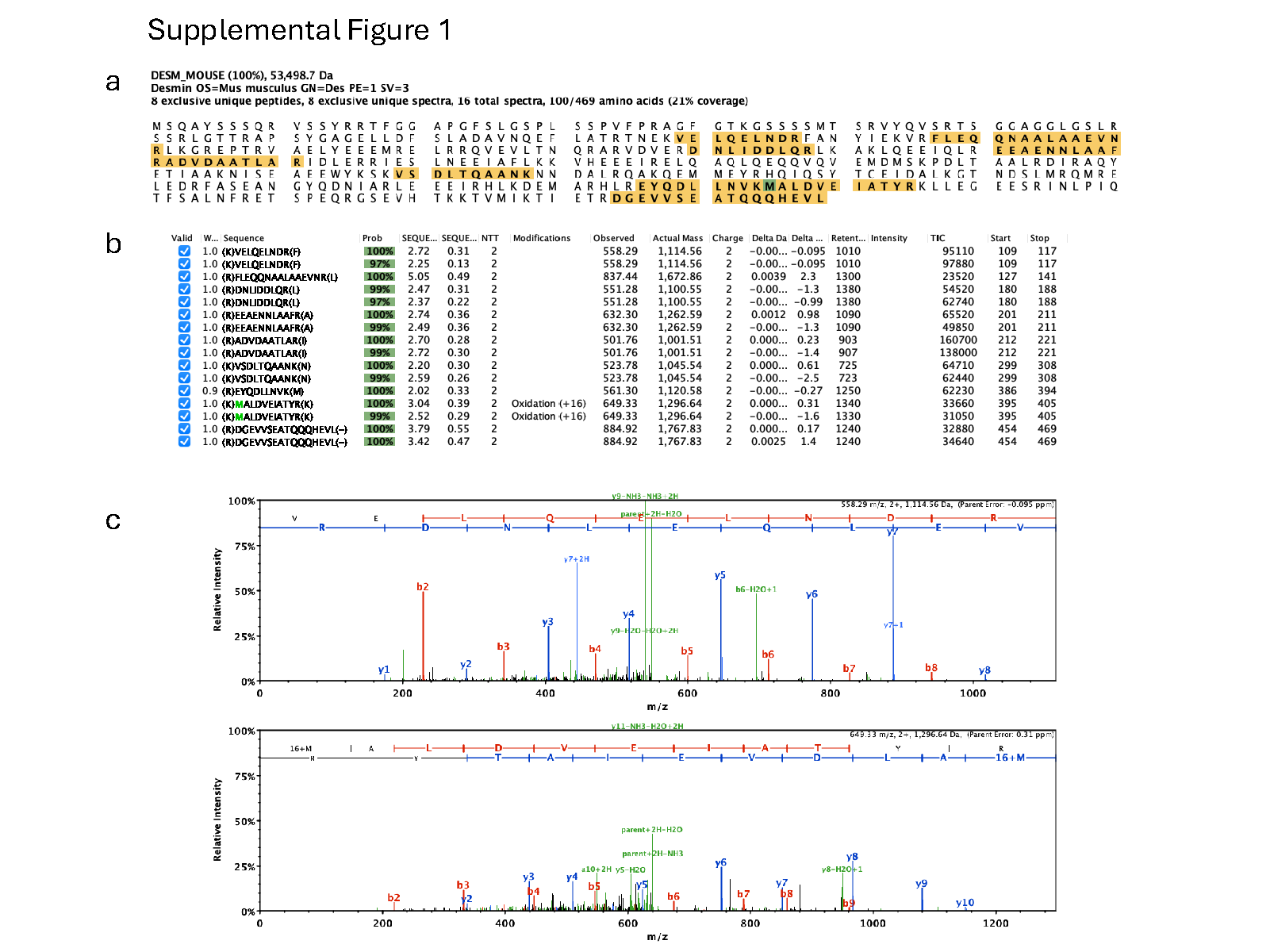

Supplement: Supplementary file 1 [file Image1.tiff]

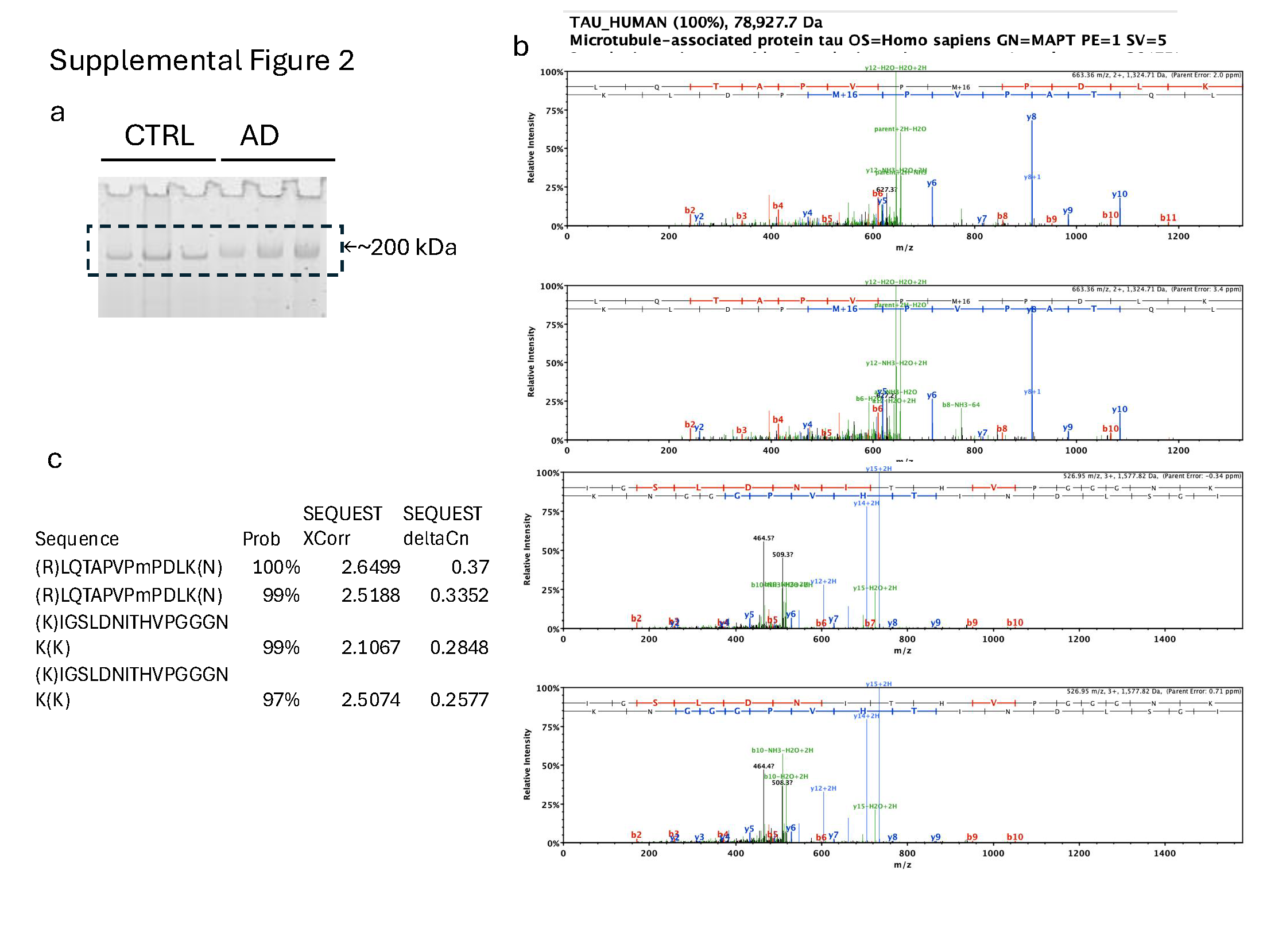

Supplement: Supplementary file 3 [file Image2.tiff]
