# Supplementary material for: Thioflavin T in-gel staining for ex vivo analysis of cardiac amyloid
Source: Front Mol Biosci. 2025 May 13;12:1505250. doi: 10.3389/fmolb.2025.1505250 (PMC12106040; doi:10.3389/fmolb.2025.1505250)
Supplement: Supplementary file 2 [file Supplementaryfile1.docx]

**Supplementary Methods and Results**

In-gel digestion was performed on bands which were located using Coomassie as a reference as previously described^16^. Liquid chromatography and tandem MS analysis (LC-MS/MS) were performed using an Ultimate 3000 nano LC (Thermo Scientific) connected to an Orbitrap Elite mass spectrometer (Thermo Scientific) equipped with an EasySpray ion source. Peptides were first loaded onto a trap column (PepMap100 C18, 5 µm, 100 Å, 300 µm i.d. x 5 mm, Thermo Scientific) followed by separation on a PepMap RSLC C18 column (2 µm, 100 Å, 75 µm i.d. x 25 mm, Thermo Scientific) using a flow rate of 300 nL/min with a linear gradient of 5-30%B for 30 minutes, 35-95%B for 3 minutes, holding at 95%B for 7 minutes and re-equilibrating at 5%B for 25 minutes at 400 nL/min (mobile phase A was 0.1% formic acid in water and mobile phase B was 0.1 % formic acid in acetonitrile). The nano-source capillary temperature was set to 275 °C and the spray voltage was set to 2 kV. MS1 scans were acquired in the Orbitrap Elite at a resolution of 60,000 FWHM (400-1700 m/z) with an AGC target of 1x106 ions over a maximum of 250 ms. MS2 spectra were acquired for the top 15 ions from each MS1 scan in CID mode in the ion trap and a target setting of 1x104 ions, an accumulation time of 100 ms, and an isolation width of 2 Da. The normalized collision energy was set to 35% and one MicroScan was acquired for each spectrum. Monoisotopic precursor selection was enabled and only MS1 signals exceeding 500 counts triggered the MS2 scans, with +1 and unassigned charge states not being selected for MS2 analysis. Dynamic exclusion was enabled with a repeat count of 2, repeat duration of 30 seconds and exclusion duration of 90 seconds. Tandem mass spectra were extracted by Proteome Discoverer (Thermo). All MS/MS samples were analyzed using Sequest (Thermo Fisher Scientific, San Jose, CA, USA; version 1.0). Sequest was set up to search the respective species-specific databases(mouse and human, uniprot) assuming the digestion enzyme with trypsin. Sequest was searched with a fragment ion mass tolerance of 1.00 Da and a parent ion tolerance of 50 PPM. Carbamidomethyl of cysteine was specified in Sequest as a fixed modification. Oxidation of methionine, phosphorylation of serine, threonine and tyrosine, glyGly of lysine and leuArgGlyGly of lysine were specified in Sequest as variable modifications.

Scaffold (version Scaffold_5.1.2, Proteome Software Inc., Portland, OR) was used to validate MS/MS based peptide and protein identifications. Peptide identifications were accepted if they could be established at greater than 95.0% probability by the Peptide Prophet algorithm (Keller, A et al Anal. Chem. 2002;74(20):5383-92) with Scaffold delta-mass correction. Protein identifications were accepted if they could be established at greater than 95.0% probability and contained at least 2 identified peptides. Protein probabilities were assigned by the Protein Prophet algorithm (Nesvizhskii, Al et al Anal. Chem. 2003;75(17):4646-58). Proteins that contained similar peptides and could not be differentiated based on MS/MS analysis alone were grouped to satisfy the principles of parsimony. Proteins sharing significant peptide evidence were grouped into clusters.

**Legends to Supplemental Figures**

**Supplemental Figure 1**

ThT-positive gel bands at ~200 kDa from the gel depicted in Figure 2c (NTG-R120G) were subjected to in-gel digestion as described. Overall sequence coverage for desmin is represented in (**a**), while the observed peptides are listed in (**b**). To representative MS spectra fro the doubluy charged peptides VELQELNDR and M*+16*ALDVEIATYR are provided in (**c**).

**Supplemental Figure 2**

ThT-positive gel bands at ~200 kDa from the gel depicted in Figure 2c (CTRL and AD, **a**), were subjected to in-gel digestion as described. Four representative spectra for peptide belonging to tau are provided (**b**) along with their sequence and score details (**c**). Of note, tau was only detected in AD samples.
